# *MINI SEED 2* (*MIS2*) Encodes a Receptor-like Kinase that Controls Grain Size and Shape in Rice

**DOI:** 10.1186/s12284-020-0368-9

**Published:** 2020-01-31

**Authors:** Yan Chun, Jingjing Fang, Syed Adeel Zafar, Jiangyuan Shang, Jinfeng Zhao, Shoujiang Yuan, Xueyong Li

**Affiliations:** 1 National Key Facility for Crop Gene Resources and Genetic Improvement, Institute of Crop Science, Chinese Academy of Agricultural Sciences, Beijing, 100081 China; 2 Shandong Rice Research Institute, Jinan, 250100 China

**Keywords:** Rice, Grain size and shape, Spikelets, Epidermal cells, Receptor-like kinase, CRINKLY4, *OsCR4*, *MIS2*

## Abstract

**Background:**

Grain size is a key agronomic trait that is directly associated with grain yield in rice. Although several genes related to grain size in rice have been identified, our understanding of the mechanism of grain development is still limited.

**Results:**

In this study, we reported the characterization of a novel seed size mutant *mini seed 2* (*mis2*), in which the grain showed reduced length, width and thickness along with wrinkled surface. Microscopic analysis revealed that the spikelet epidermal cell size was reduced but the cell number was increased in the *mis2* mutant, suggesting that *MIS2* controls grain size by coordinately regulating epidermal cell size and cell number. Map-based cloning revealed that *MIS2* encodes a receptor-like kinase CRINKLY4 (CR4) which showed the highest expression in developing panicles. The MIS2 protein is localized primarily on the plasma membrane along with the endosome. However, the Arg258Gln mutation located in extracellular domain in the *mis2* mutant disturbed its subcellular localization. Additionally, three major haplotypes of *MIS2* were identified in the *japonica*, *indica* and *aus* rice cultivars. The 18-bp InDel (insertion and deletion) in the 5′-UTR (untranslated region) caused different expression level of *MIS2* in haplotypes.

**Conclusions:**

We reported a key role of *OsCR4* in controlling grain size and shape by coordinately regulating epidermal cell size and cell number. The Arg258 in the extracellular seven-repeat domain is essential for the correct subcellular behavior and function of the OsCR4 protein.

## Background

Rice (*Oryza sativa L*.) is one of the most important grain crops providing food for over half of the world’s population. To meet the demand of the increasing population, rice yield needs to increase up to 50% by 2030 (Ahmadi et al. 2014). Number of panicles per plant, number of grains per panicle, grain weight and size are key determinants of final grain yield in rice (Huang et al. 2013; Zafar et al. 2019). Grain size and weight usually have positive correlation with each other as grain weight normally depends on grain size (Tan et al. 2000). Thus, identification of genes controlling these key agronomic traits has been a promising goal of rice genetic improvement (Jiang et al. 2019a).

Several quantitative trait loci (QTL) have been reported that are linked with grain size and shape in rice, thus contributing toward breeding high yielding rice. However, only a few genes have been functionally characterized (Nan et al. 2018). Most of these genes regulate grain size and shape by affecting grain filling, cell number and cell size. *GIF1* (*GRAIN INCOMPLETE FILLING 1*) encodes a cell-wall invertase, regulating rice grain filling and grain weight. Ectopic expression of the cultivated *GIF1* gene with the *CaMV35S* or rice *Waxy* promoter resulted in smaller grains, whereas overexpression of *GIF1* driven by its own promoter increased grain size (Wang et al. 2008). Many studies reveal that the number of cells in the hull of spikelet plays an important role in determining grain size (Xu et al. 2018a, 2018b). *GW2*, encoding a RING-type protein with E3 ubiquitin ligase activity, controls rice grain width and weight by regulating number of cells in spikelet (Song et al. 2007). Similarly, loss of function of *GSN1* (*GRAIN SIZE AND NUMBER 1*), which encodes a mitogen-activated protein kinase phosphatase (OsMKP1), caused larger grains by increasing cell number due to enhanced cell division during spikelet development (Guo et al. 2018). XIAO, a putative LRR receptor-like kinase, is known to regulate organ size including leaves, panicles and grains via controlling number of cells while the cell size was constant (Jiang et al. 2012). Besides cell number, the cell size and shape are also important determinants of grain size. *GL7* (*GRAIN LENGTH ON CHROMOSOME 7*)/*GW7*, a major QTL for rice grain length and width, encodes a protein homologous to *Arabidopsis thaliana* LONGIFOLIA protein and regulates spikelets longitudinal cell elongation (Wang et al. 2015a; Wang et al. 2015b). The longer and narrower spikelet hulls of NIL-GL7 plants were the result of an increase in cell length and a decrease in cell width of epidermal cells of the outer and inner glumes (Wang et al. 2015a). *GLW7*, encoding the plant-specific transcription factor OsSPL13, regulates grain length and thickness but does not regulate grain width. Loss of function of *GLW7* causes reduced size of outer parenchyma cells, but the number of cells remains unchanged (Si et al. 2015). In some instances, the grain size and shape are controlled by more than one factor at the same time. A putative serine carboxypeptidase GS5 positively controls grain size by regulating both grain width and grain filling. In the two near-isogenic lines (NILs) of GS5, the wider grains contain more number and large size of parenchyma cells (Li et al. 2011). GSE5 (GRAIN SIZE ON CHROMOSOME 5) is a plasma membrane-associated protein with IQ domains, which interacts with the rice calmodulin protein, OsCaM1–1. Loss of *GSE5* function caused wide and heavy grains due to more and narrower spikelet epidermal cells in the mutant (Duan et al. 2017). These studies suggest that the rice grain shape and size are mainly determined by cell number and size.

Plant receptor-like kinases (RLKs) are among the largest protein super families with diverse extracellular domains that are linked with a conserved kinase domain via transmembrane part (Pu et al. 2017). The extracellular domain functions as the major sensor module at the cell surface that regulates multiple biological pathways. The RLKs protein activation generally occurs in response to ligand binding of extracellular domain and subsequently, the downstream signaling is mediated by phosphorylation of the cytoplasmic kinase domain (Cock et al. 2002; Tichtinsky et al. 2003). The CRINKLY4 (CR4) family RLKs in angiosperms are known to regulate epidermal cell differentiation (Becraft et al. 1996; Watanabe et al. 2004; Pu et al. 2012). The first *cr4* mutant was reported in maize which produced crinkled leaves and aleurone-defective kernels (Becraft et al. 1996). In *Arabidopsis*, loss of function of *ACR4* led to the phenotypes including abnormal texture in integuments and seed coat, and reduced hydrophobicity on leaf surface (Gifford et al. 2003; Watanabe et al. 2004; Cao et al. 2005). The *cr4* mutants also showed abnormalities in seed shape and differences in seed size (Gifford et al. 2003). In rice, knock-down of *OsCR4* by RNA interference produced spikelets with separated palea and lemma, as a result of tumour-like cells in the outer epidermis and wart-like cells in the inner epidermis (Pu et al. 2012). However, the function of *OsCR4* in the regulation of rice grain size is still not clear.

In the present study, we isolated a small seed mutant “*mini seed 2* (*mis2*)” in rice that displayed smaller grains with irregular shape. The *mis2* mutant spikelet had reduced cell size but increased cell number, which suggests *MIS2* regulates grain size through coordinate regulation of cell size and cell number. The candidate gene *MIS2* encodes the receptor-like kinase OsCR4 and the mutation in the *mis2* caused the disturbed subcellular behavior of MIS2 protein. Moreover, we identified three major haplotypes from over 500 core rice germplasm. Our study provides a new perspective of *OsCR4* function in regulating grain size and shape in rice.

## Methods

### Plant Materials and Growth Conditions

The *mis2* mutant was isolated from ethyl methane sulfonate (EMS)-treated seeds of the elite *japonica* variety, XuDao3 (XD3). For genetic analysis and map-based cloning, the *mis2* mutant was crossed with the *indica* variety Dular. All rice plants were cultivated in paddy fields in Beijing, Shandong and Hainan provinces under natural growth conditions.

### Phenotypic Analysis Using Simple and Electron Microscopy

Photographs of plants and panicles were taken using a Digital camera (Nikon). Photographs of mature seeds (with and without hulls) were taken using stereomicroscope (Olympus). For cross section analysis, the young spikelets of WT and the mutant were fixed in FAA (50% ethanol, 5% glacial acetic acid, and 5% formaldehyde) overnight at 4 °C, then dehydrated in a graded ethanol series as described previously (Zafar et al. 2019). After fixing with xylene, the samples were embedded in paraplast (Sigma). The embedded samples were sliced into 8–10 μm thick sections with a rotary microtome (Leica). Sections were dewaxed in xylene, hydrated through a graded ethanol series, stained with 1% fast green and observed under a light microscope (Olympus).

For scanning electron microscopy (SEM), the spikelets at maturity were fixed in 3.5% glutaraldehyde solution and then dehydrated through an ethanol series. After dehydration process, the samples were dried by critical-point drying method and sputter-coated with aurum, and then observed under the scanning electron microscope (Hitachi). Cell length and cell width of spikelets were measured using Image J software.

For transmission electron microscopy (TEM), the young spikelets before heading stage were fixed by 2.5% glutaraldehyde solution (PH 7.2) and vacuum-infiltrated. The samples were washed three times with 0.2 mol/L sodium cacodylate buffer for 30 min, fixed in 10% osmic acid for 1 h, washed three times with deionized water for 45 min, dehydrated with ethanol and treated with acetone. Samples were then embedded in epoxy resins and polymerized at 70 °C and then cut into about 500–800 Ǻ thin sections and stained by the mixture of uranyl acetate dihydrate and led citrate. The sections were washed with deionized water and visualized using HITACHI Transmission Electron Microscope (HT7700).

### Map-Based Cloning of *MIS2*

F_2_ mapping population was generated by crossing *mis2* with *indica* variety Dular. Primary mapping was conducted with InDel markers by 60 F_2_ mutant individuals. To fine-map the *MIS2* locus, new molecular markers were developed. *MIS2* was mapped to a 213-kb region on chromosome 3, and genes from this region were amplified and sequenced from both *mis2* and the WT. The primer sequences for the InDel markers are showed in Additional file 1: Table 1.

### Vector Construction and Plant Transformation

To make the genomic DNA complementation construct, the 5997-bp genomic DNA fragment of *MIS2* including 2867-bp upstream of start codon and 425-bp downstream of stop codon was amplified from the WT and cloned into *EcoRI* and *PmlI* sites of the plant binary vector *pCAMBIA1305.1*. For the promoter-GUS vector, a 2870-bp fragment upstream of the *MIS2* start codon was amplified from WT and cloned into the *pCAMBIA1305.1* vector to generate the plasmid ProMIS2:GUS. The constructs after sequencing were transformed into rice calli by *Agrobacterium tumefaciens* mediated transformation. In-Fusion HD Cloning Kit (Clontech) was used in this study to produce various DNA constructs. The PCR primers are given in Additional file 1: Table 1.

### RNA Extraction and Quantitative Real-Time PCR

Total RNA was extracted from various plant organs of the WT using RNAprep Pure Plant Kit (TIANGEN) and was reverse transcribed using HiScript II Q RT SuperMix (Vazyme). Quantitative real-time PCR analysis was performed on an Applied Biosystems 7500 Real-Time PCR System with 2 × ChamQ SYBR Color qPCR Master Mix (Vazyme) and the data were calculated using the 2^−∆∆Ct^ quantification method. The rice Ubiquitin gene was used as an internal control to normalize gene expression. All primers used are listed in Additional file 1: Table S1.

### GUS Staining

The histochemical GUS activities were detected as described previously (Jefferson et al. 1987). The samples were vacuum-infiltrated for 30 min in GUS staining buffer. After overnight incubation in dark at 37 °C, the samples were cleared using ethanol to remove chlorophyll and then photographed using stereomicroscope (Olympus).

### Subcellular Localization of MIS2

To determine the subcellular localization of normal and mutated versions of MIS2, the coding region of *MIS2* and *MIS2*^*mu*^ were amplified from WT and the *mis2* mutant cDNA, and fused with GFP at C-terminus, respectively. The coding region of Ara6 was amplified from *Arabidopsis* Col-0 and fused with mCherry at C-terminus. *MIS2* and *MIS2*^*mu*^ were driven by rice actin1 promoter, and *Ara6* was driven by CaMV35S promoter. The constructs were introduced into *Agrobacterium tumefaciens* strain GV3101 and infiltrated into the *Nicotiana benthamiana* leaves. The GFP and mCherry fluorescence in leaf epidermal cells was detected with the LSM 510 META confocal lasers scanning microscope (Zeiss). The sequences of the PCR primers used for vector construction are given in Additional file 1: Table 1.

### Bioinformatics Analysis

The three-dimensional modeling of the MIS2 extracellular crinkly repeats was undertaken based on the x-ray structures of photoreceptor AtUVR8 mutant W285F and light-induced structural changes at 120 K (Zeng et al. 2015). The protein shares 18.75% pairwise identity with the query sequence (Arnold et al. 2006; Benkert et al. 2011; Biasini et al. 2014). The structure was assigned by the standard settings within PyMol 2.2.0 (https://pymol.org). Alignment was conducted by Clustal W and phylogenetic analysis was performed using MEGA X (Kumar et al. 2018). GENEDOC was used to show the consensus and shading.

### Haplotype Analysis

The genotype data of the 524 accessions was obtained via the 3 K Rice Genomes Project from NCBI (https://www.ncbi.nlm.nih.gov/sra/?term=PRJEB6180) (Wang et al. 2018). The alleles of low frequency in the 524 panels were considered missing, and the heterozygous alleles were also eliminated.

## Results

### Phenotypic Characterization of the *mis2* Mutant

**Fig. 1:**
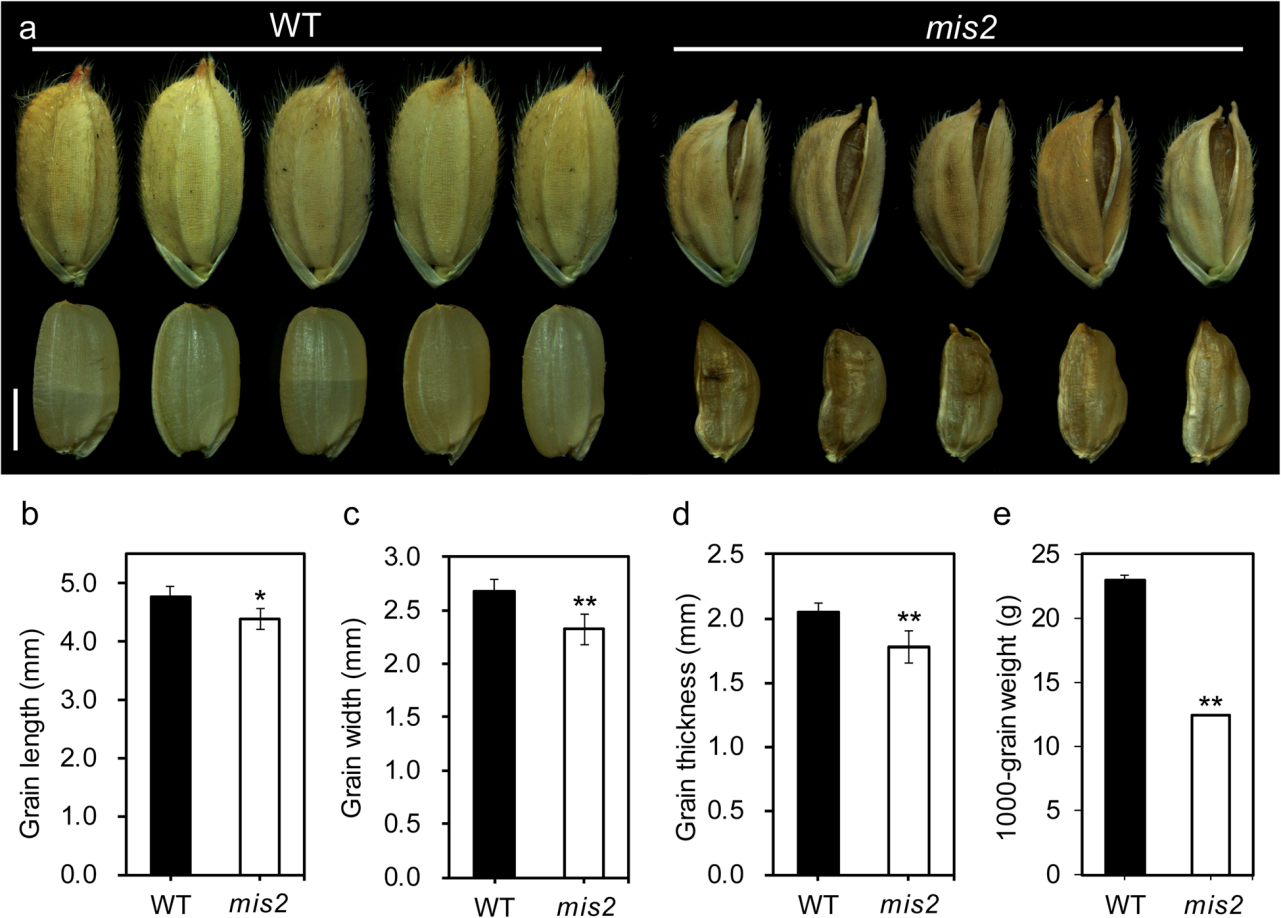
Morphological characterization of the *mis2* grains. (**a**) Mature grains from WT and *mis2*. Upper: unhulled grains; Lower: hulled garins. Scale bar = 2 mm. (**b-e**) Quantification data of grain length (**b**), grain width (**c**), grain thickness (**d**) and 1000-grain weight (**e**) of WT and *mis2*. * *P* < 0.05 and ** *P* < 0.01 by Student’s *t* test. Data are given as mean ± SD (*n* = 15)

The *mis2* mutant was isolated from the M_2_ population of *japonica* cv. XuDao 3 (XD3) mutagenized by ethyl methane sulphonate (EMS). Compared with the wild type (WT), the *mis2* mutant has small grains with irregular shape and open glumes (Fig. 1a). The grain size including grain length, width, thickness and 1000-grain weight was significantly reduced in the *mis2* mutant as compared to WT (Fig. 1b-e). In addition to the grain size and shape, several other agronomic traits were also affected in the *mis2* mutant. The *mis2* mutant was shorter in height and produced more tillers per plant (Additional file 1: Figure S1a, d, e). In addition, the seed setting rate was also decreased in the *mis2* compared with the WT, while the spikelet number per panicle was increased (Additional file 1: Figure S1 g, h). However, the panicle length was not changed significantly between the WT and *mis2* mutant (Additional file 1: Figure S1b, c, f). These observations indicated that *MIS2* has significant contribution in determining the various seed morphology related characters and controls seed size in rice.

### *mis2* Affects Grain Size by Coordinately Regulating the Epidermal Cell Size and Cell Number

**Fig. 2:**
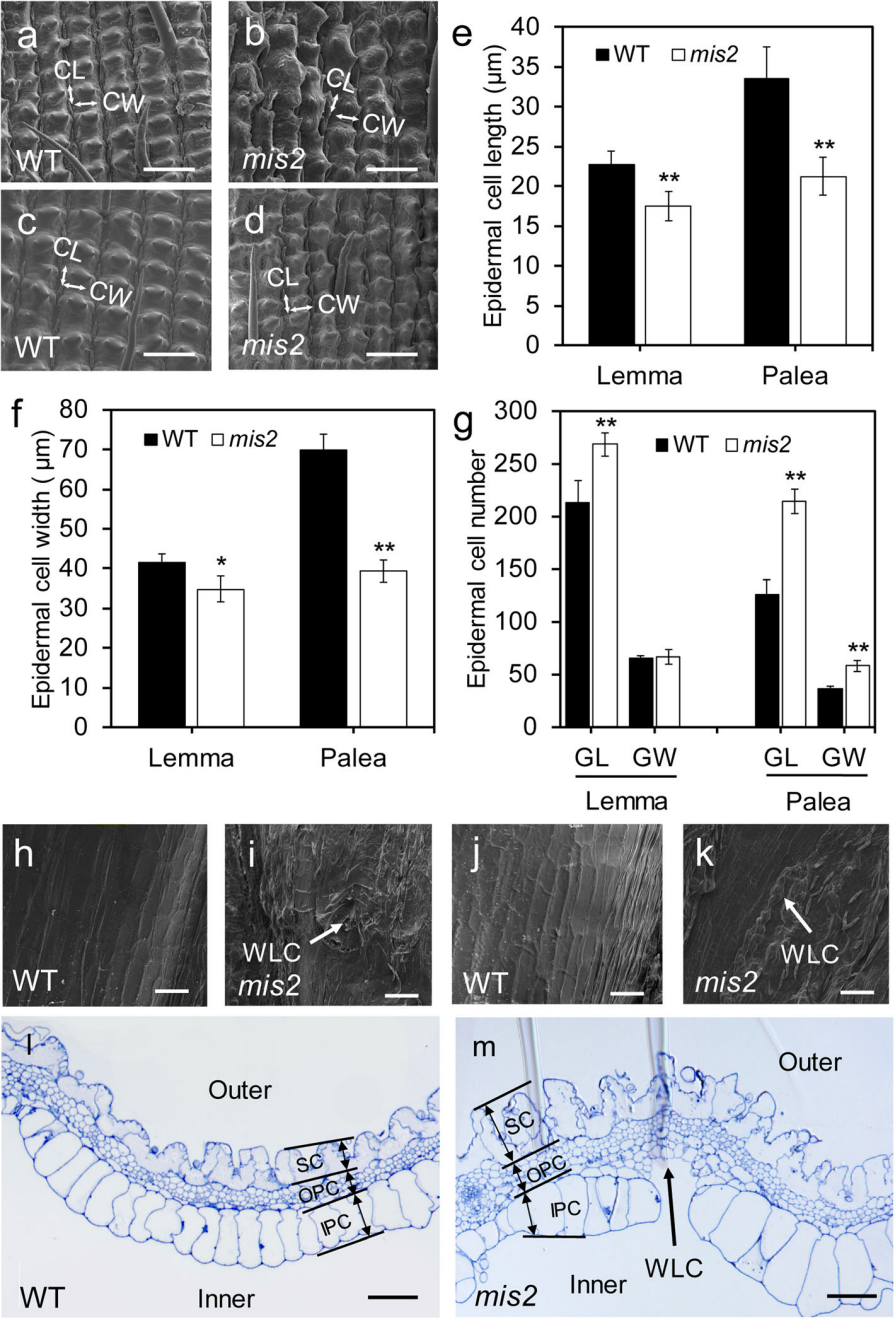
Comparison of the spikelet epidermal cells between WT and *mis2*. (**a**-**d**) Scanning electron micrographs (SEM) for the outer surfaces of lemma (**a**, **b**) and palea (**c**, **d**) of WT (**a**, **c**) and *mis2* (**b**, **d**). CL and CW indicate epidermal cell length and cell width orientation, respectively. Bars = 100 μm. (**e**, **f**) The average length and width of the epidermal cells. (*n* = 10). (**g**) Comparisons of the calculated epidermal cell number of the lemma and palea in the grain-length (GL) and grain-width (GW) direction, respectively (*n* = 10). (**h**-**k**) SEM for the inner surfaces of lemma (**h**, **i**) and palea (**j**, **k**) of WT and *mis2*. Bars = 100 μm. (**l**, **m**) Cross sections of WT and *mis2* spikelet hull. Bar = 50 μm. WLC, wart-like cells; SC, silicified cells; OPC, outer parenchyma cells; IPC, inner parenchyma cells. * *P* < 0.05 and ** *P* < 0.01 by Student’s *t* test. Data are given as mean ± SD (*n* = 20)

To reveal the cellular basis of smaller grain size in the *mis2* mutant, both outer and inner surfaces of mature lemmas and paleas of the WT and the *mis2* mutant were observed by scanning electron microscopy (SEM). In the WT, regular and linear arrangement of epidermal silicified cells was observed in the outer epidermal surface of the lemma and palea (Fig. 2a, c). In contrast, the epidermal cells in the *mis2* mutant were wrinkled with variable shape and size (Fig. 2b, d). More detailed observations indicated that the length and width of the epidermal cells was significantly reduced in the *mis2* mutant as compared with WT (Fig. 2e, f). However, the epidermal cell number of the lemma and palea was increased to different extents in both grain-length and grain-width directions (Fig. 2g). Regarding the inner surface, the epidermis of the lemma and palea of the WT was smooth, whereas in the *mis2* mutant, it was uneven and produced wart-like structures (Fig. 2h-k). Taken together, these observations demonstrated that MIS2 is a positive regulator of grain size and shape and coordinately regulates spikelet epidermal cell size and number. Cross section observations of the lemma and palea further validated our SEM results. We observed a regular pattern of three cell layers including silicified cells (SC), outer parenchyma cells (OPC) and inner parenchyma cells (IPC) in the WT (Fig. 2l). However, the epidermal silicified cells in the *mis2* mutant were unclear and disordered (Fig. 2m). The cells of the OPC layer were also irregular and more in number in the *mis2* mutant as compared with WT (Fig. 2l, m). We also observed remarkable difference in the cells of the IPC layer among WT and *mis2*. The cells of IPC layer were uniform in shape and size in WT while those of *mis2* were of irregular shape and variable size (Fig. 2l, m). The IPC layer was also discontinuous in some locations in the *mis2* mutant.

**Fig. 3:**
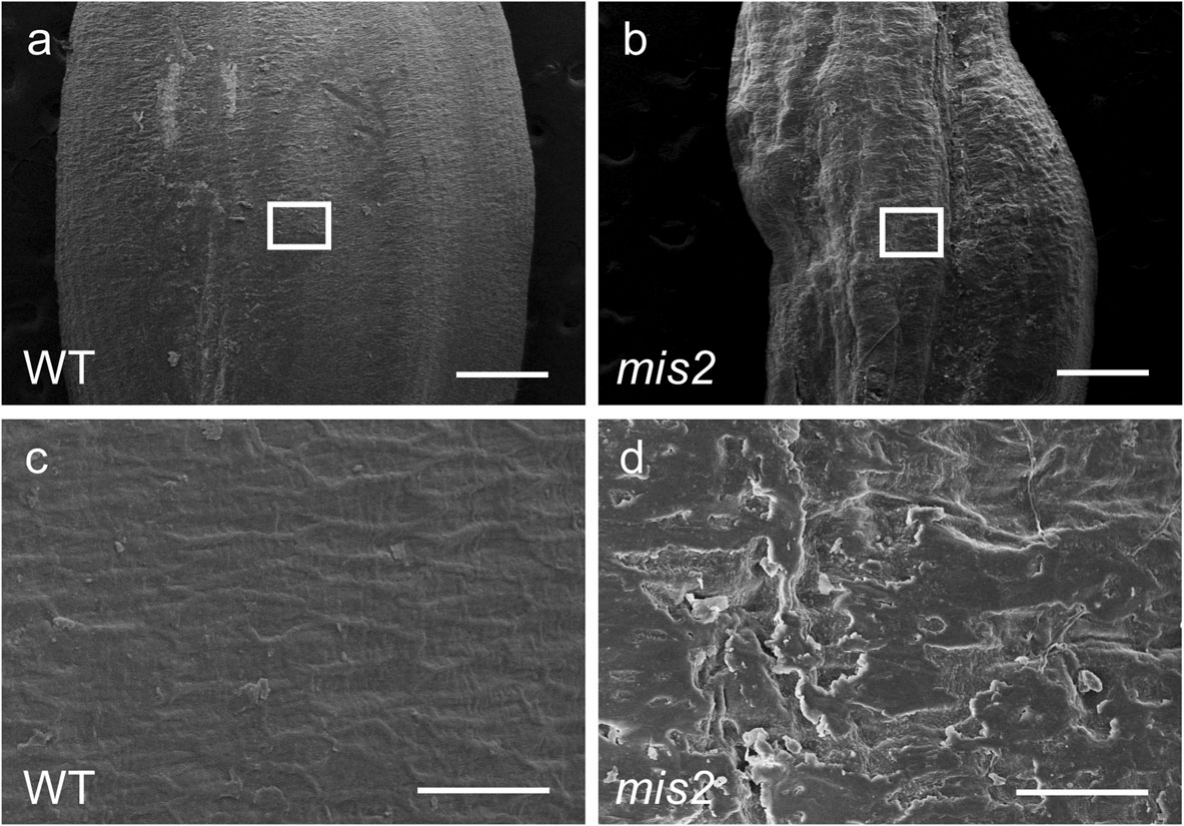
Morphological comparison of seed surface between WT and *mis2*. (**a**, **b**) SEM for the surface of WT and *mis2* seed. Bar = 0.5 mm. (**c**, **d**) Magnified views of the boxed region in (**a**, **b**), respectively. Bar = 50 μm

We also observed the grain surface of the WT and *mis2* mutant using SEM. This observation displayed smooth epidermis layer in the WT while rough and wrinkled epidermal layer in the *mis2* mutant (Fig. 3a, b). A closer observation indicated that grain epidermis was full of cracks in the *mis2* as compared with WT (Fig. 3c, d). These findings suggest that *MIS2* also plays role in determining grain shape and surface in rice.

### The *mis2* Mutant Has Defective Interlocking Structures and Epidermal Cell Wall

**Fig. 4:**
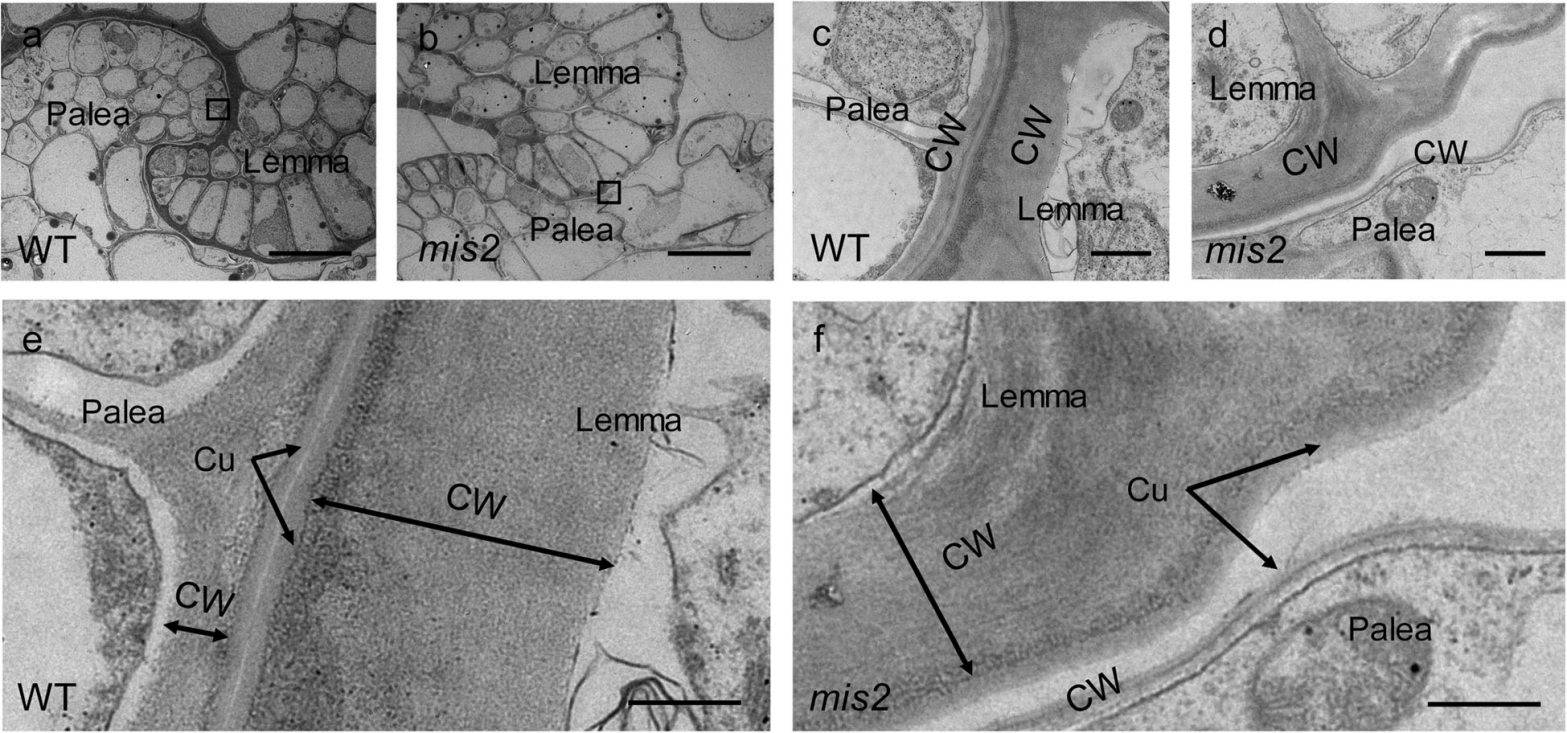
Comparison of the interlocking cells, cell wall and cuticle layer between WT and *mis2*. (**a**, **b**) Transmission electron micrographs (TEM) for the interlocking cells of the palea and lemma in the WT and *mis2*. Bar =10 μm. (**c, d**) Magnified views of the boxed region in (**a**, **b**), respectively. Bar =5 μm. (**e**, **f**) Cuticle and cell wall of interlocking cells in the WT and *mis2*. Bar =1 μm. Cu, cuticle; CW, cell wall

The lemma and palea of the WT spikelets were interlocked tightly, whereas the *mis2* mutant displayed open-hull spikelets (Fig. 1a). To analyze this abnormal phenotype, the interlocking structures were investigated by transmission electron microscopy (TEM). In the WT, two hook-like structures were tightly interlocked but the interlocking structure was defective in the *mis2* mutant (Fig. 4a, b). Although, there is no obvious difference in interlocking cells of lemma, however, the cells at the joint in the palea were fewer than those in the WT and showed amorphous shape, leading to the disappearance of hook-like structure (Fig. 4b).

In the WT, the epidermal cells at the interlocking position were covered with an even cell wall, and the cell wall was fused with a clear cuticle layer (Fig. 4c, e). In the *mis2* mutant, however, the cell wall of those cells was curved and fused with a thin cuticle layer which was either uneven or not as clear as that in the wild type (Fig. 4d, f). These observations suggest that the *mis2* mutant had defective development of cell wall and cuticle layer of interlocking cells in the lemma and palea.

### *MIS2* Encodes the Receptor-like Kinase OsCR4

**Fig. 5:**
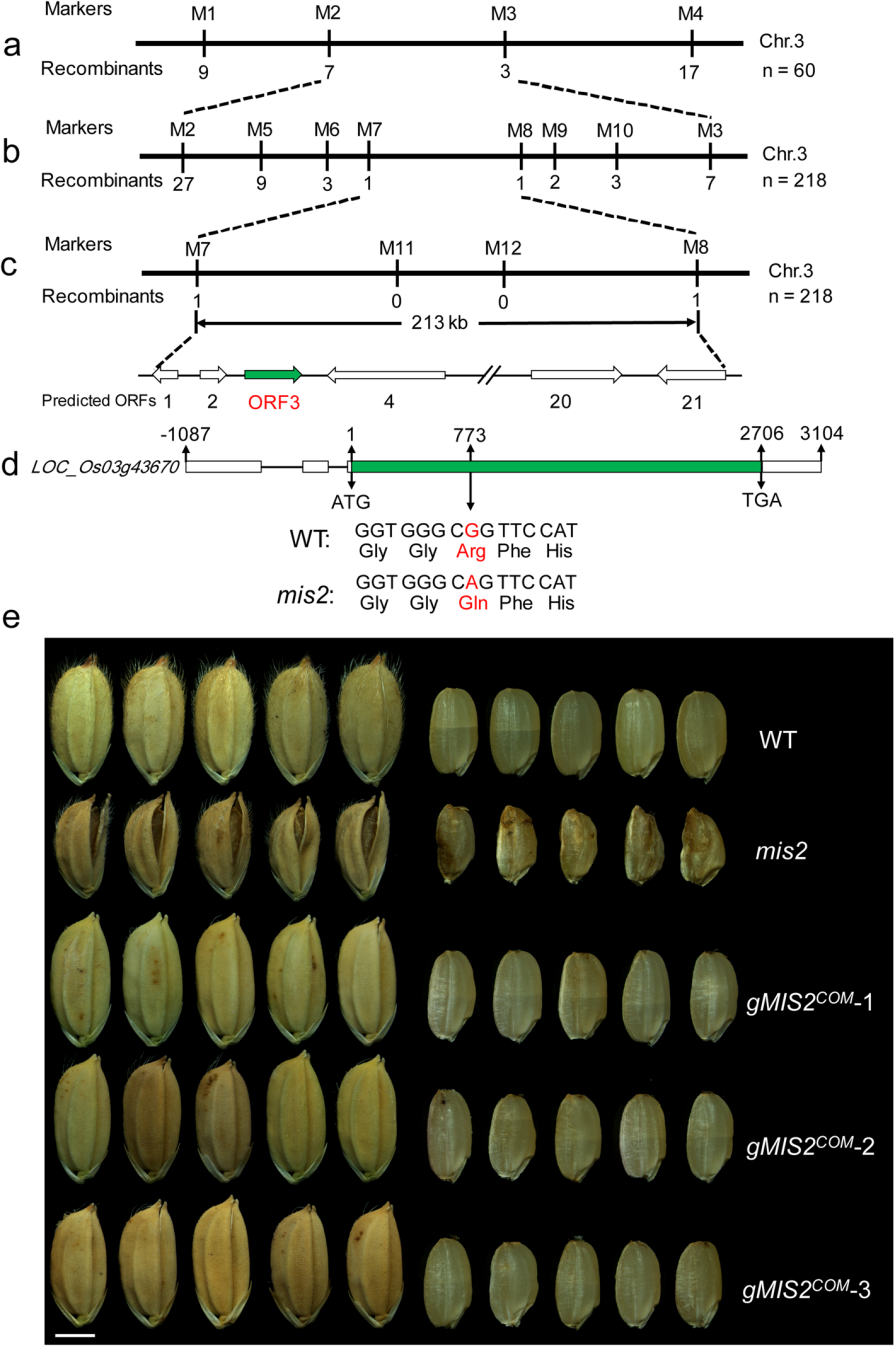
Map-Based cloning of *MIS2* and genetic complementation. (**a**) The *MIS2* locus was mapped to a region between markers M2 and M3 on chromosome 3. (**b**, **c**) The candidate gene was further delimited to a 213-kb genomic region between markers M7 and M8. Twenty-one candidate genes are predicted in this region. The numbers beneath the marker positions indicate the number of recombinants. (**d**) The *MIS2* gene structure. White and green box represent untranslated region and coding sequence, respectively. Black lines represent introns. The start codon (ATG) and the stop codon (TGA) are indicated. A single nucleotide mutation from G to A in *MIS2* resulted in an arginine-to-glutamine change. (**e**) Complementation of mature grain morphology. Three genomic DNA complementation transgenic lines are shown. Bar = 2.5 mm

To know the causal gene underlying the *mis2* phenotype, the *mis2* mutant was crossed with the WT cv. XD3. All the F_1_ plants had normal grains as WT. The F_2_ population was segregated in a 3:1 ratio generating 276 WT-like plants having normal grains and 85 *mis2*-like plants having small and wrinkled grains (χ^2^ = 0.407, χ^2^_0.05, 1_ = 3.84). This indicated that the *mis2* mutant phenotype was regulated by a single recessive gene. Using 60 F_2_ mutant plants from a cross between the *mis2* mutant and the *indica* variety Dular, the *MIS2* gene was primarily mapped on chromosome 3 between two insertion-deletion (InDel) markers M2 and M3 (Fig. 5a). For fine mapping, 14 InDel markers were developed and the *MIS2* locus was narrowed to a 213-kb region by a F_2_ population consisting of 218 mutant plants. This region contained 21 open reading frames (ORFs) based on MSU Rice Genome Annotation Project (http://rice.plantbiology.msu.edu/) (Fig. 5b, c), including 13 functional proteins, three expressed proteins and five retrotransposons.

To determine the candidate gene, 16 genes encoding functional or expressed protein were amplified and sequenced from both the WT and *mis2* mutant genomic DNA. A single-nucleotide substitution in the *mis2* mutant was detected in the *ORF3* (*LOC_Os03g43670*) which contains 3 exons, and no mutation was found in other 15 genes. According to the annotation on MSU Rice Genome Annotation Project, *ORF3* encodes the putative receptor-like kinase CRINKLY4 (OsCR4) which contains a seven-repeat structure and Ser (serine)/ Thr (threonine) kinase domain. The mutation from G to A was detected on the third exon, causing the replacement of 258th amino acid arginine (R) with glutamine (Q) (Fig. 5d). The R258Q substitution in the *mis2* mutant occurred on the sixth repeat of OsCR4 (Additional file 1: Figure S2). To verify whether *ORF3* was responsible for the *mis2* mutant phenotype, the complete genomic DNA of *ORF3* including its own promoter (around 1.8 kp upstream of ATG) was transformed into the *mis2* mutant. Seven transgenic lines carrying the *ORF3* fragment were obtained and all of them fully complemented the mutant phenotype having large grains with smooth surface and closed-hulls (Fig. 5e). This data confirmed that *ORF3*, which is referred to as *MIS2* hereafter, was the candidate gene for the *mis2* mutant phenotype.

To know the evolutionary relationship of MIS2 with other CR4 family proteins, a phylogenetic analysis was conducted using MEGA-X version 10.0.1. The CR4 family and their related proteins in *Arabidopsis*, rice and maize fell into two major clusters. The first cluster contained two subclades, one having only *Arabidopsis* CRR proteins and other subclade having only CR4 proteins from *Arabidopsis*, rice and maize (Fig. S3). The other cluster contained only CRR proteins from different species. Notably, no ortholog of AtCRR1 and AtCRR2 in rice and maize was found (Additional file 1: Figure S3).

### Expression Pattern of *MIS2*

**Fig. 6:**
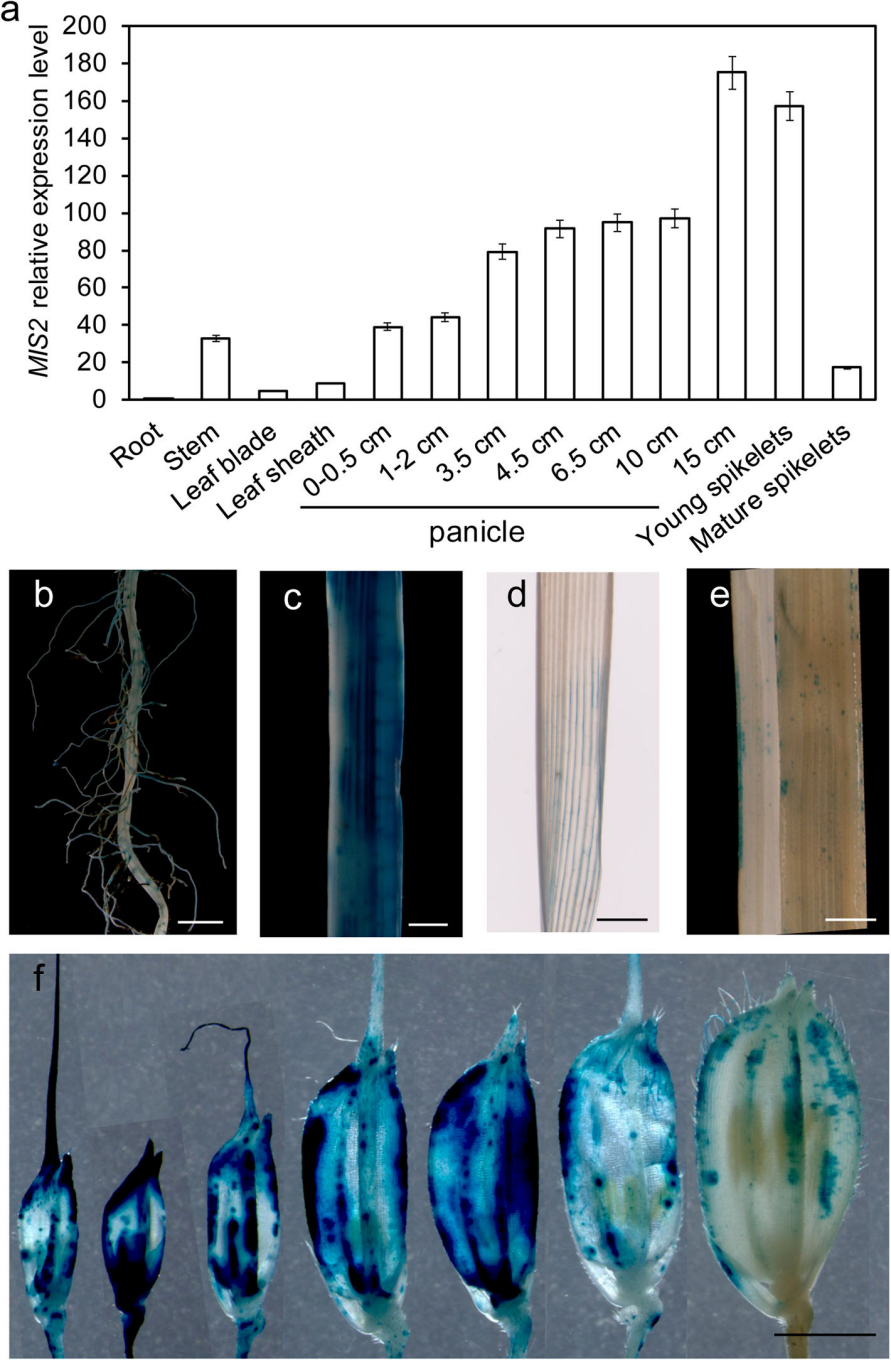
Expression pattern of *MIS2.* (**a**) Quantitative RT-PCR analysis showing the relative expression level of *MIS2* in root, stem, leaf blade, leaf sheath, young panicle at various length stage, young spikelets and mature spikelets. The rice ubiquitin gene was used as an internal control. Values are given as mean ± SD (*n* = 3). (**b**-**f**) Expression pattern of *GUS* gene driven by the *MIS2* promoter in root (**b**), stem (**c**), leaf blade (**d**), leaf sheath (**e**) and spikelets at different stages (**f**). Bar = 2 mm

To examine the spatio-temporal expression pattern of *MIS2,* quantitative real-time PCR (qRT-PCR) and histochemical promoter-*GUS* staining approaches were used. The qRT-PCR analysis indicated that the expression level of *MIS2* was relatively higher in panicle tissues than other organs (Fig. 6a). Notably, the expression was gradually increased in developing panicles with the highest expression in panicle at 15-cm length stage which is corresponding to the anthesis stage. After that, it started to decrease in mature panicle (Fig. 6a).

The results of promoter-GUS assay were more or less consistent with qRT-PCR. Histochemical staining of transgenic plants indicated low expression in leaf and roots as compared with stem (Fig. 6b-e) and highest expression in developing panicles (lemma and palea) (Fig. 6f). These results also suggested that MIS2 functions mainly during the spikelets development.

### *mis2* Disrupted the Distribution and Recycle of OsCR4

**Fig. 7:**
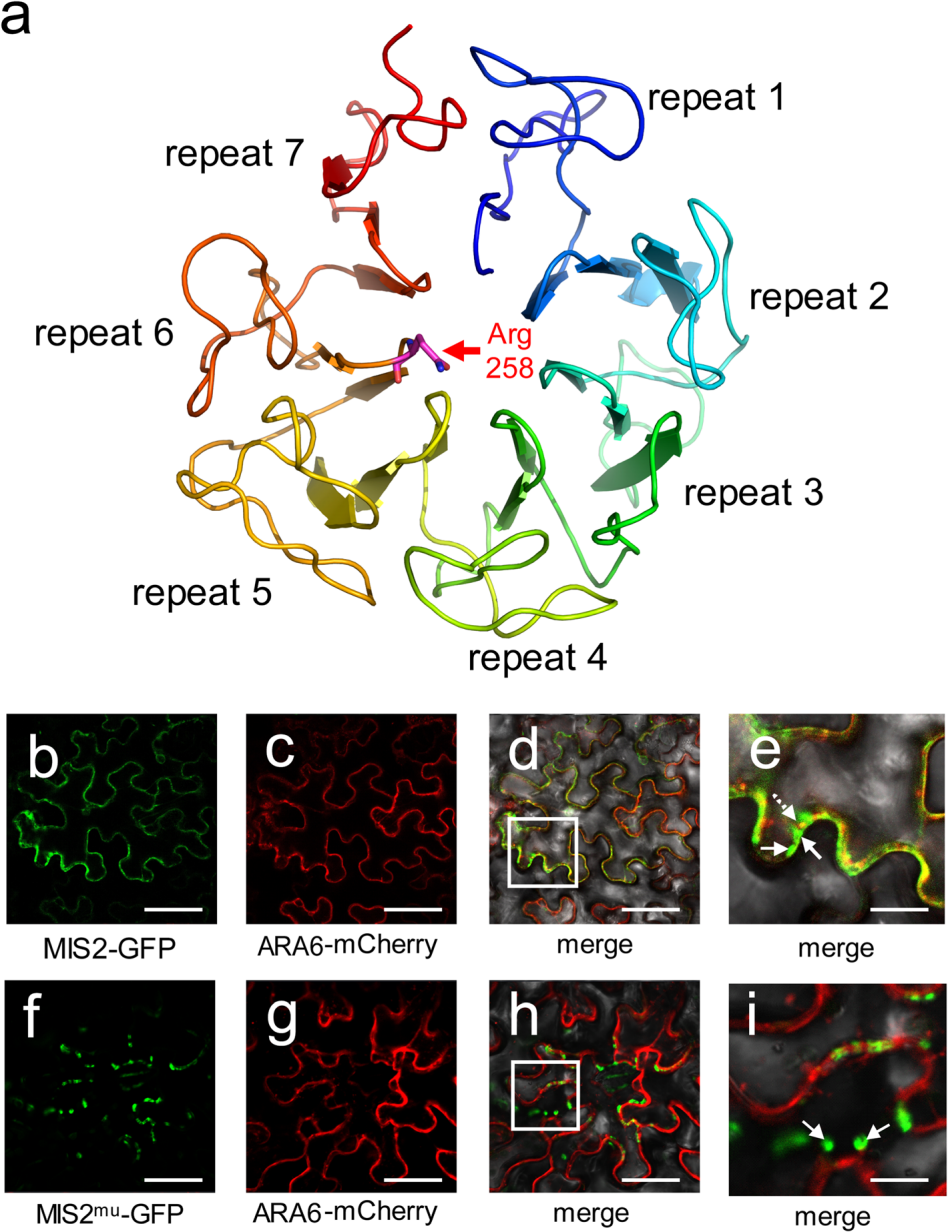
Subcellular behavior of MIS and MIS2^mu^ protein. (**a**) Three-dimensional model of the MIS2 extracellular repeat domain using PyMol. Seven repeats are labeled and Arg 258, which is mutated to Gln in *mis2*, is shown as a stick and colored purple. (**b**-**e**) Co-expression of *MIS2* and *ARA6* in tobacco leaf. The florescence of MIS2::GFP (**b)** and ARA6::mCherry (**c**) were detected and merged on the plasma membrane (**d**). The merged image (**e**) is a magnification view of the boxed region in (**d**). Line arrows indicate GFP-only vesicles, and dot arrows indicate co-localizations. Bar = 20 μm (**b**-**d**), Bar = 10 μm (**e**). (**f**-**i**) Co-expression of MIS2^mu^ and ARA6 in tobacco leaf epidermal cells. ARA6::mCherry is localized on plasma membrane (**g**). MIS2^mu^::GFP localization is considerably changed (**f**, **h**). Magnification view shows GFP-labeled and mCherry-labeled compartments in cytoplasm and no colocalization is observed (I). Line arrows indicate GFP-only vesicles, respectively. Bar = 20 μm (**f**-**h**), Bar = 10 μm (**i**)

OsCR4 encoded by the *MIS2* gene is one of the typical receptor-like kinases of the TNFR (Tumor Necrosis Factor Receptor-like) subfamily. OsCR4 contains a seven-repeat extracellular domain (Fig. 7a), a tumor necrosis factor receptor domain, a single transmembrane helix and an intracellular Ser/Thr kinase domain (Additional file 1: Figure S2). It is known that ACR4, the ortholog of OsCR4 in *Arabidopsis*, is localized on plasma membrane and endosomes (Giffhord et al. 2005; Tian, et al. 2007). Meanwhile, ACR4 protein can be internalized and turned over through two distinct endosomes in *Arabidopsis* root cells, representing a population of internalized vesicles and protein export bodies. It is also known that the extracellular seven-repeat domain is required for the ACR4 function, internalization and turnover (Giffhord et al. 2005; Qin et al. 2019).

In the *mis2* mutant, the R258Q substitution occurred on the sixth repeat of the extracellular domain (Fig.7a and Additional file 1: Figure S2). To see whether this mutation affected the subcellular behavior of MIS2 protein, the coding sequence (CDS) of *MIS2* from WT and the *mis2* mutant were fused with GFP and co-expressed with *ARA6* (*At3g54840*) which was fused with mCherry, respectively, in tobacco (*Nicotiana benthamiana*). ARA6 is a plant-unique Rab5 GTPase in *Arabidopsis* which mediates trafficking from endosomes to the plasma membrane but counteracts endocytic trafficking from the endosomes to the vacuoles (Ebine et al. 2011; Tsutsui et al. 2015). ARA6 is localized to the plasma membrane and small punctate structures in the early step of the endocytic pathway (Ueda et al. 2001; Ebine et al. 2011). The green signals of MIS2::GFP were observed in the plasma membrane and could merge with ARA6::mCherry (Fig. 7b-d). Meanwhile, some cells have shown the presence of two distinct populations of GFP-containing bodies, one merged with mCherry (Fig. 7e, dot arrow) and the other only with GFP (Fig. 7e, line arrow), representing protein export bodies and internalized vesicles, respectively. This indicated that the subcellular behavior of MIS2 protein is similar to ACR4, which could be internalized and rapidly turned over. In contrast, the mutated version of MIS2 (MIS2^mu^::GFP) was localized in the cytoplasm near the plasma membrane but could not merge with ARA6:mCherry (Fig. 7f-h). A few large GFP-containing bodies were observed in cytoplasm representing the internalized vesicles (Fig. 7i, line arrow). However, none of these vesicles merged withARA6:mCherry, suggesting that the internalized protein can not be exported and recycled to the membrane. Thus, we concluded that the R258Q substitution in the extracellular domain disrupted the distribution and turnover of MIS2 protein in the *mis2* mutant, leading to the insufficient replenishment of OsCR4 onto the plasma membrane.

### *mis2* Affected the Expression of Genes Related to Epidermal Differentiation, Grain Size and BRs

**Fig. 8:**
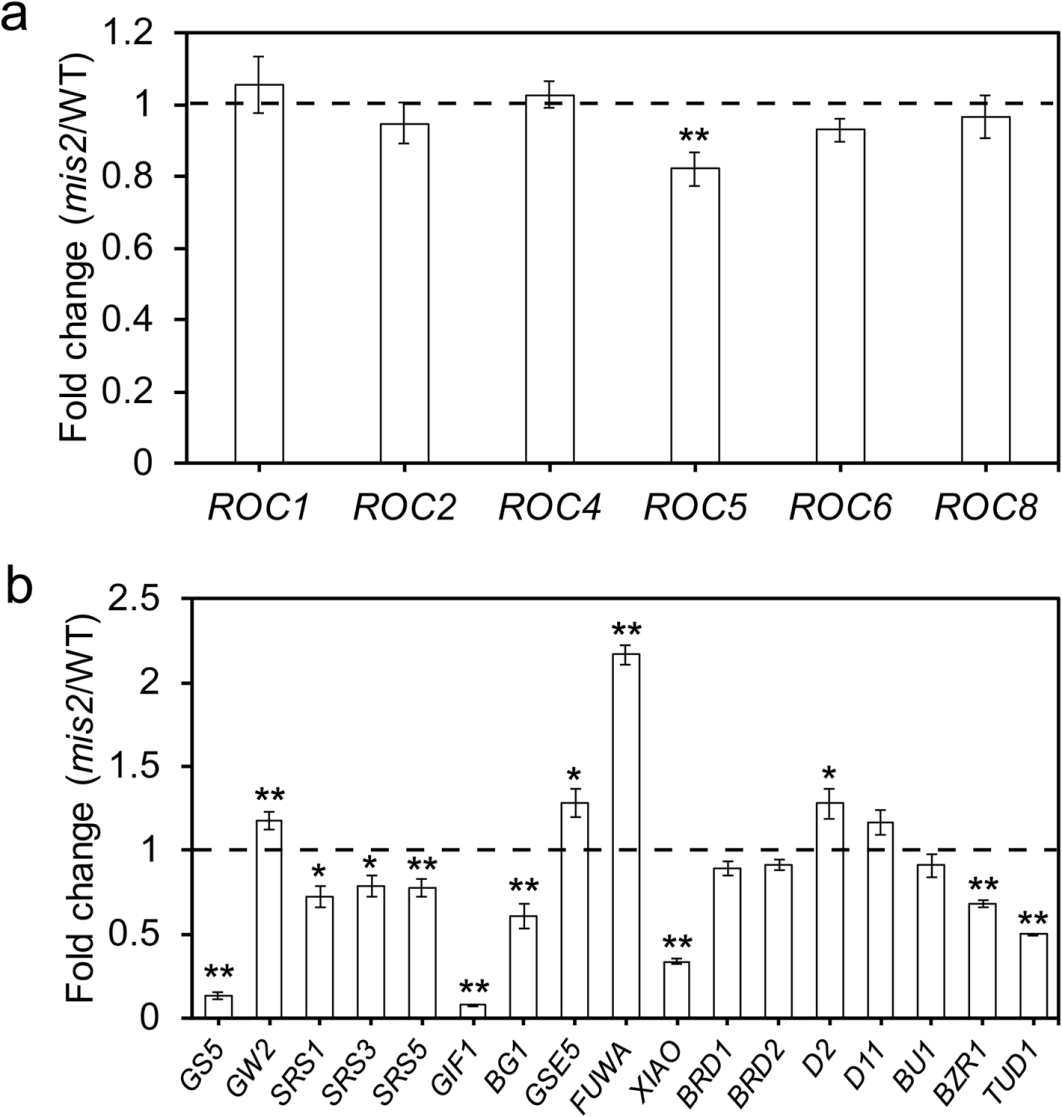
Quantitative RT-PCR analysis of epidermal cell development, grain size and BRs related genes. (**a**) Relative expression fold change of *ROC5* and related genes in WT and *mis2*. (**b**) Relative expression fold change of genes related to grain size and BRs in WT and *mis2*. Young inflorescence of ~ 1 cm was used for analysis. Rice ubiquitin gene was used as an internal control. **P < 0.01 and *P < 0.05 by Student’s *t* test. Values are given as mean ± SD (*n* = 3)

The expression of *ACR4* in *Arabidopsis* is regulated by HD-ZIP class IV transcription factors *Arabidopsis thaliana* MEIRISTEM LAYER 1 (ATML1) and PROTODERMAL FACTOR 2 (PDF2) in a negative feedback model (San-Bento, et al. 2014). ATML1 and PDF2 positively or negatively regulate *ACR4* expression via the L1 box (Tanaka, et al. 2002; Abe, et al. 2003; San-Bento, et al. 2014), and *ACR4* positively regulates epidermal cell differentiation upstream of *ATML1* (Tanaka et al. 2007). The ortholog of *ATML1* and *PDF2* in rice is *Rice outermost cell-specific gene 5* (*ROC5*) and thus the relative expression level of *ROC5* and other paralogous genes in young panicle (~ 1 cm) was examined in the WT and *mis2* mutant (Fig. 8a). *ROC5* has essential roles in the formation and development of epidermal bulliform cells in rice, and the *ROC5* T-DNA insertion knockout mutant had significantly increased bulliform cell number and size, producing the abaxially rolled leaf (Zou et al., 2011). However, the role of *ROC5* in regulating rice grain size and the spikelet epidermal cells development is not known.

To investigate the possible regulatory relationship between *MIS2* and other previously identified genes that are involved in rice grain size and shape, the transcript levels of some grain size and shape related genes were detected (Song et al. 2007; Abe et al. 2010; Kitagawa et al. 2010; Li et al. 2010; Li et al. 2011; Jiang et al. 2012; Segami et al. 2012; Chen et al. 2015; Liu et al. 2015; Duan et al. 2017;). The expression level of *GIF1* which positively regulates grain filling was significantly decreased (Fig. 8b). *SRS3* and *SRS5* which positively control the spikelet epidermal cell size or shape were significantly down regulated in the *mis2* mutant (Fig. 8b). The genes which positively regulate the spikelet epidermal cell number including *GS5* and *XIAO* were also down regulated, whereas the negative regulators *GW2* and *FUWA* were up regulated (Fig. 8b). Additionally, the expression levels of genes which control both cell size and cell number were significantly changed such as *GSE5*, *SRS1* and *BG1* (Fig. 8b). Brassinosteroids (BRs) are a class of vital phytohormones and their roles in the regulation of seed size have been well reported (Mori et al. 2002; Tanaka et al. 2009; Bai et al. 2007; Hu et al. 2013; Zhang et al. 2014; Song. 2017; Tong and Chu. 2018; Hong et al. 2002). The BRs signalling-related genes including *XIAO*, *BZR1* and *TUD1* were down regulated, while BRs synthesis genes *D2* and *D11* were slightly up regulated (Fig. 8b). These results suggested that *MIS2* may act as an upstream regulator in rice grain development by affecting the expression of various genes, and *MIS2* probably have a complex relationship with BRs.

### Haplotype Analysis of *MIS2* in Diverse Germplasm

**Fig. 9:**
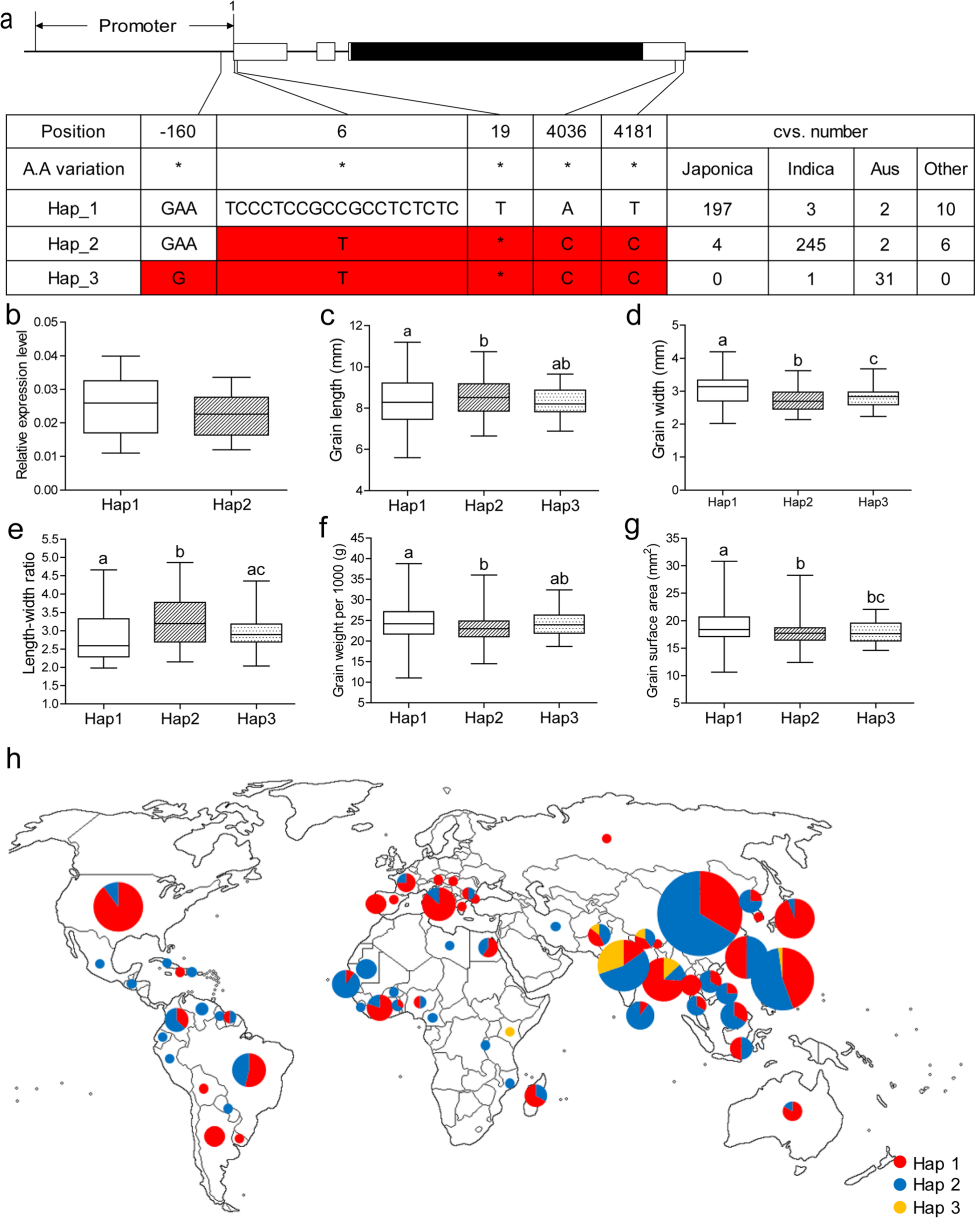
Haplotype and origin analysis of *MIS2*. (**a**) *MIS2* gene structure and natural variations among 524 rice accessions. (**b**) Relative expression level of Hap1 and Hap 2 in young panicle. (**c**-**g**) Comparison of grain length (**c**), grain width (**d**), length-width ratio (**e**), grain weight per 1000 (**f**) and grain surface area (**g**) among accessions containing Hap1, Hap2 and Hap3. The letter on histogram (**a**, **b** and **c**) indicate significant differences (*P* < 0.05) by ANOVA. (H) Geographic origin of accessions containing Hap1, Hap2 and Hap3. Hap1, Hpa2 and Hap3 are represented by red, blue and yellow circles, respectively

To analyze the variation of *MIS2* in natural population, nucleotide polymorphism in the promoter and gene body region of *MIS2* was investigated among 524 diverse rice germplasm including *japonica*, *indica*, *aus* and other subspecies selected from the 3000 Rice Genomes Project (Wang et al. 2018). Excluding heterozygotes and low frequency variations, three haplotypes (Hap1, Hap2 and Hap3) were classified by single nucleotide polymorphisms (SNPs) and InDels (Fig. 9a). Interestingly, the variations in coding region were all synonymous mutations, and potential functional SNPs and InDels were only detected in promoter and untranslated regions (UTR). This indicated that the coding sequence (CDS) of *MIS2* was highly conserved in cultivars and the natural mutations in CDS region may easily cause unfavorable phenotype and thus be obsoleted during breeding selection. Hap1 were represented mainly by accessions from the *japonica* subpopulation, containing 197 *japonica*, three *indica* and two *aus* cultivars. This genotype preference was also found in Hap2 and Hap3 which had high frequency in *indica* and *aus* subpopulation, respectively (Fig. 9a). Notably, compared with Hap1, an 18-bp deletion in 5′-UTR of *MIS2* was detected in Hap2 and Hap3. To check the possible effect of this InDel on the *MIS2* gene expression, transcript level of Hap1 and Hap2 were examined using mRNA prepared from young panicles. The result showed that Hap1 had a bit higher expression level than Hap2 (Fig. 9b).

To investigate the phenotypic differences among the three major haplotypes, grain length, grain width, length-width ratio, 1000-grain weight and grain surface area were examined (Fig. 9c-g). The accessions carrying Hap1 displayed wider, higher 1000-grain weight and larger surface of the grain but shorter and lower length-width ratio than the accessions of Hap2, whereas the Hap3 accessions were intermediate. Geographical analysis showed that Hap1 accessions which were represented by *japonica* varieties were mainly distributed in northern areas, whereas Hap2 and Hap3, or *indica* and *aus* accessions were mainly located in southern regions (Fig. 9h). This suggested that Hap1 may have a different origin area from Hap2 and Hap3.

## Discussion

Rice is consumed as a staple food by more than half of the world’s population (Huo et al. 2017; Zafar et al. 2018). Grain size and weight are the key quantitative traits affecting final grain yield in rice. Several genes that regulate grain size or shape have been identified in rice such as *GS3*, *GS5*, *GSE5*, *GW2*, *SRSs* and *BG1* (Song et al. 2007; Tanabe et al., 2007; Abe et al., 2010; Kitagawa et al. 2010; Mao et al. 2010; Li et al., 2011; Segami et al., 2012; Duan et al. 2017). However, our understanding of the genetic mechanism of grain size development is still limited (Li and Li., 2016). In this study, we report a novel grain size mutant named “*mis2”* which displayed smaller grains and irregular spikelet structure (Figs. 1 and 2). Cytological observations indicated that the *mis2* mutant produced smaller but more epidermal cells in the lemma and palea (Fig. 2e-g), suggesting that *MIS2* plays a contrasting role in regulating cell size and cell number. Interestingly, several other seed size mutants or near-isogenic lines (NILs) of rice and *Arabidopsis* also showed this coordinate regulation phenomenon such as *gse5*, NIL-*TGW3*, *da1* and *da2* (Xia et al. 2013; Duan et al. 2017; Ying et al. 2018). The *mis2* mutant also produced other phenotypes such as open-hull and irregular epidermal cell wall (Figs. 1, 2). Map-based cloning identified *MIS2*/*OsCR4* as a candidate gene for *mis2* phenotype which encodes a Ser/Thr receptor-like kinase.

Several *CR4* family members have been identified and characterized in different plant species including *Arabidopsis*, maize, and *Physcomitrella patens* (Becraft et al. 1996; Gifford et al. 2003; Demko et al. 2016). *CR4* in maize affects leaf epidermis differentiation. *Mu* transposon insertional mutants of *CR4* produced wrinkle leaves and inhibited seed aleurone formation (Becraft et al. 1996). Similar phenotypes were also found in EMS and chromosome breakage mutants (Jin et al. 2000; Becraft et al. 2001). The *Arabidopsis acr4* mutants which contained T-DNA insertions produced abnormal epidermal cell differentiation in the seed, leaf, ovule integument, sepal margin and endothelium (Gifford et al. 2003; Watanabe et al. 2004; Cao et al. 2005). The *PpCR4* deletion mutant in *Physcomitrella patens* generated crinkly phyllids and smaller sporophytes due to lack of differentiated margin cells and abnormal epidermal cells, respectively (Demko et al. 2016). These mutants suggest that *CR4* family play a vital role in epidermal cell development. Notably, *OsCR4* knock-down lines created by RNA interference (RNAi) revealed the essential role of *OsCR4* in maintaining the interlocking of the lemma and palea by promoting epidermal cell differentiation (Pu et al. 2012). However, no any loss-of-function mutant of *OsCR4* have been reported in rice until now. In this study, by using a loss-of-function mutant of *OsCR4*, we report for the first time a key role of *OsCR4* in controlling grain size and shape by coordinately regulating epidermal cell size and cell number.

RLKs are characterized with an extracellular domain that serves as an important sensor molecule (Gifford et al. 2005). However, studies are required to further elucidate the mechanism of its function. Here we demonstrated that the substitution at R258Q in the sixth repeat of extracellular domain disrupted the subcellular behavior of MIS2 protein in the *mis2* mutant (Fig. 7). MIS2 is mainly a plasma membrane-localized protein with some internalized vesicles. However, the R258Q mutation in MIS2 extracellular domain has changed its localization from plasma membrane to cytoplasm, suggesting a key role of extracellular domain in subcellular localization (Fig. 7). The subcellular behavior of nonfunctional MIS2^mu^::GFP was different from that of the ACR4^C180Y^::GFP, which was localized normally to the plasma membrane (Gifford et al. 2005). Meanwhile, deleting 4.5 of seven repeats of ACR4 led to a more stable protein. Export of ACR4 protein to the membrane was normal, but the internalization was disturbed (Gifford et al. 2005). These various subcellular behaviors indicate the diversity and complexity of CR4 family protein localization and function. As plant RLKs, the fate of CR4 family protein following the internalization is still not clear. In animals, some receptor kinases such as the EGFR family have been demonstrated to undergo ubiquitination. Ubiquitination seems to play an important role in both receptor internalization and deciding whether the internalized receptor will be degraded or recycled back to the membrane (Dikic and Giordano, 2003; Marmor and Yarden, 2004). Therefore, we proposed that the substitution R258Q of MIS2 probably affect the ubiquitination signal, hence increasing the rate of internalized protein degradation and diminishing recycling back to the plasma membrane. This recycle of the protein was insufficient for the supply at plasma membrane. Additionally, it has been reported that the seven-bladed β-propeller structure mediates diverse functions especially in protein-protein and protein-ligand interactions (Chen, et al. 2011). Whether the mutation of R258Q affect the binding between MIS2 and ligand, or break the homo-oligomerization or hetero-oligomerization of MIS2 with other proteins, which may influence the subsequent fate of MIS2, needs to be investigated by further studies.

HD-Zip IV transcription factors ATML1 and PDF2 play a redundant and essential role in the *Arabidopsis* embryo development via a universally active feedback loop (San-Bento et al. 2014; Ogawa et al. 2015). ACR4 can activate the signalling pathway of ATML1 and PDF2, resulting in dimer formation between ATML1 and PDF2. In turn, these dimers repress *ACR4* and their own transcription (San-Bento et al. 2014). The *atml1–3 pdf2–4* double mutant displayed arrested embryos development, abnormal shoot epidermal cells and pale aborted seeds (Ogawa et al. 2015). To check whether there is a similar mechanism in rice, transcript level of *ATML1* and *PDF2* orthologs (*ROC* genes) in rice were detected. Among the six *ROC* genes, only *ROC5* was significantly downregulated in the *mis2* mutant (Fig. 8a), suggesting that *ROC5* may act downstream of *MIS2*. *ROC5* is known to regulate leaf epidermal bulliform cells formation in rice. Knockout mutant of *ROC5* produced out-curved leaves, longer panicle, more spikelets per panicle and lower seed-setting rate (Zou et al. 2011). However, there is no study on the role of *ROC5* in controlling seed shape or seed epidermal cells development. This suggests that different members of the *ROC* family could have redundant function in seed development.

To see if *MIS2* regulate the expression of other seed size related genes, we measured the transcript level of several reported genes. Genes including *SRS1*, *SRS3, SRS5, GW2*, *FUWA*, *GS5*, *XIAO*, *GSE5* and *BG1* have been reported to control epidermal cell size or number in rice (Song et al. 2007; Abe et al. 2010; Li et al. 2010; Kitagawa et al. 2010; Li et al. 2011; Zou et al. 2011; Jiang et al. 2012; Segami et al. 2012; Chen et al. 2015; Liu et al. 2015; Duan et al. 2017). The expression of all these genes was significantly altered in the *mis2* mutant (Fig. 8), suggesting that *MIS2* may function upstream of these genes and can affect the expression of these genes. This indicates a key role of *MIS2* in regulating seed size in rice via affecting epidermal cell size or number. BRs are a class of vital phytohormones and their role in the regulation of seed size is well reported (Mori et al. 2002; Tanaka et al. 2009; Bai et al. 2007; Hu et al. 2013; Zhang et al. 2014; Song. 2017; Tong and Chu. 2018; Hong et al. 2002). To see if *MIS2* participates in the BR pathway, we measured the relative expression of several BRs biosynthesis and signalling genes in WT and *mis2* (Fig. 8b). *XIAO*, *BZR1* and *TUD1* were down-regulated in the *mis2* mutant, suggesting that *MIS2* may play a role in regulating seed size via affecting the BR signalling. We also observed an up-regulation in the expression of *D2* and *D11* (two BR biosynthesis genes) which could be due to a compensatory response. Nevertheless, further confirmation studies are needed to expand our understanding about the interaction of *MIS2* and BR-related genes.

Haplotype analysis revealed that the *MIS2* gene was highly differentiated in various subgroups including *japonica*, *indica* and *aus* (Fig. 9a). The phenotype data of different haplotypes showed that grain width, 1000-grain weight and grain surface area of Hap1 was relatively higher than those in Hap2, while Hap2 had increased grain length and higher length-width ratio (Fig. 9c-g). Hap3 data was intermediate in these two haplotypes. Hap1, Hap2 and Hap3 accessions were mainly comprised of *japonica*, *indica* and *aus* respectively. Hap1 accessions were mainly distributed in northern areas, whereas Hap2 and Hap3 accessions were mainly located in southern regions, indicating that the three haplotypes may have different origins (Fig. 9h). Notably, the variations occurred in promoter and UTR regions and no missense variation in coding sequence was detected in Hap1, Hap2 and Hap3. This is probably explained by that the mutation in CDS is unfavorable for breeding. Compared with Hap1, an 18-bp fragment which contains an ERE (ethylene response elements) or GCC box was deleted from the 5′-UTR region of Hap2 and Hap3 (Fig. 9a). The GCC box is an 11- bp sequence (TAAGAGCCGCC) with a core GCCGCC sequence and was reported as the binding site of the AP2/EREBP (APETALA2/ethylene responsive element binding protein) family transcription factors (Ohme-Takagi and Shinshi., 1995; Büttner and Singh., 1997; Zhou et al., 1997; Fujimoto et al., 2000). In *Arabidopsis* and rice, *AP2* family transcription factors play an important role in determining seed size and seed weight, such as *APETALA2*, *RSR1* and *SSH1* (Ohto et al., 2005; Jofuku et al., 2005; Fu and Xue, 2010; Jiang et al. 2019b). Therefore, we hypothesize that this deletion probably abolishes the binding of certain *AP2* family transcription factor to the 5’UTR region of *MIS2*, leading to different expression level in various haplotypes (Fig. 9b).

## Conclusions

In this study, a rice *mini seed 2* (*mis2*) mutant was characterized. The smaller grain was due to the coordinate alternation in epidermal cell size and cell number, while the wrinkle surface was due to the irregular shape and variable size of epidermal cells. The *MIS2* gene was revealed to encode the receptor-like kinase CRINKLY4 (CR4) known to regulate epidermal cell differentiation. The MIS2 protein is localized primarily on the plasma membrane along with the endosome. The *mis2* mutant protein harboring the Arg258Gln substitution in extracellular domain disturbed its localization on the plasma membrane probably due to insufficient recycling of CR4 from endosome to plasma membrane. Haplotype analysis of *MIS2* in 524 diverse rice accessions revealed an 18-bp INDEL in the 5′-UTR region but no missense mutation in the coding region. This study suggests that CR4 is essential for the rice grain development and the extracellular domain is required for its proper subcellular localization and function.

## Supplementary information

**Additional file 1 Figure S1.** Phenotype of the *mi2* mutant plant. **Figure S2.** Sequence alignments of CR4 family protein from rice, maize and *Arabidopsis*. **Figure S3.** Phylogenetic analysis of CR4 family and CR4-related proteins. **Table S1.** Primers used in this study.

## Data Availability

The authors declare that all data generated or analyzed during this study are available within the manuscript or its supplementary files or are available from the corresponding authors upon request.

## Abbreviations

CDS: Coding sequence
CR4: CRINKLY4
EMS: Ethyl methane sulphonate
INDEL: Insertion and deletion
m*is2*: *Mini seed 2*
ORF: Open reading frame
RLK: Receptor-like kinase
SEM: Scanning electron microscopy (SEM)
SNP: Single nucleotide polymorphisms
TEM: Transmission electron microscopy
TNFR: Tumor necrosis factor receptor-like
UTR: Untranslated region
