# Supplementary material for: MINI SEED 2 (MIS2) Encodes a Receptor-like Kinase that Controls Grain Size and Shape in Rice
Source: Rice (N Y). 2020 Jan 31;13:7. doi: 10.1186/s12284-020-0368-9 (PMC6994593; doi:10.1186/s12284-020-0368-9)
Supplement: Supplementary file 1 — Additional file 1 Figure S1. Phenotype of the mi2 mutant plant. Figure S2. Sequence alignments of CR4 family protein from rice, maize and Arabidopsis. Figure S3. Phylogenetic analysis of CR4 family and CR4-related proteins. Table S1. Primers used in this study. [file 12284_2020_368_MOESM1_ESM.pdf]

***MINI SEED 2* (*MIS2*) Encodes a Receptor-like Kinase that Controls Grain Size and Shape in Rice**

Yan Chun[1], Jingjing Fang[1], Syed Adeel Zafar[1], Jiangyuan Shang[1], Jinfeng Zhao[1], Shoujiang Yuan[2] and Xueyong Li[1,*]

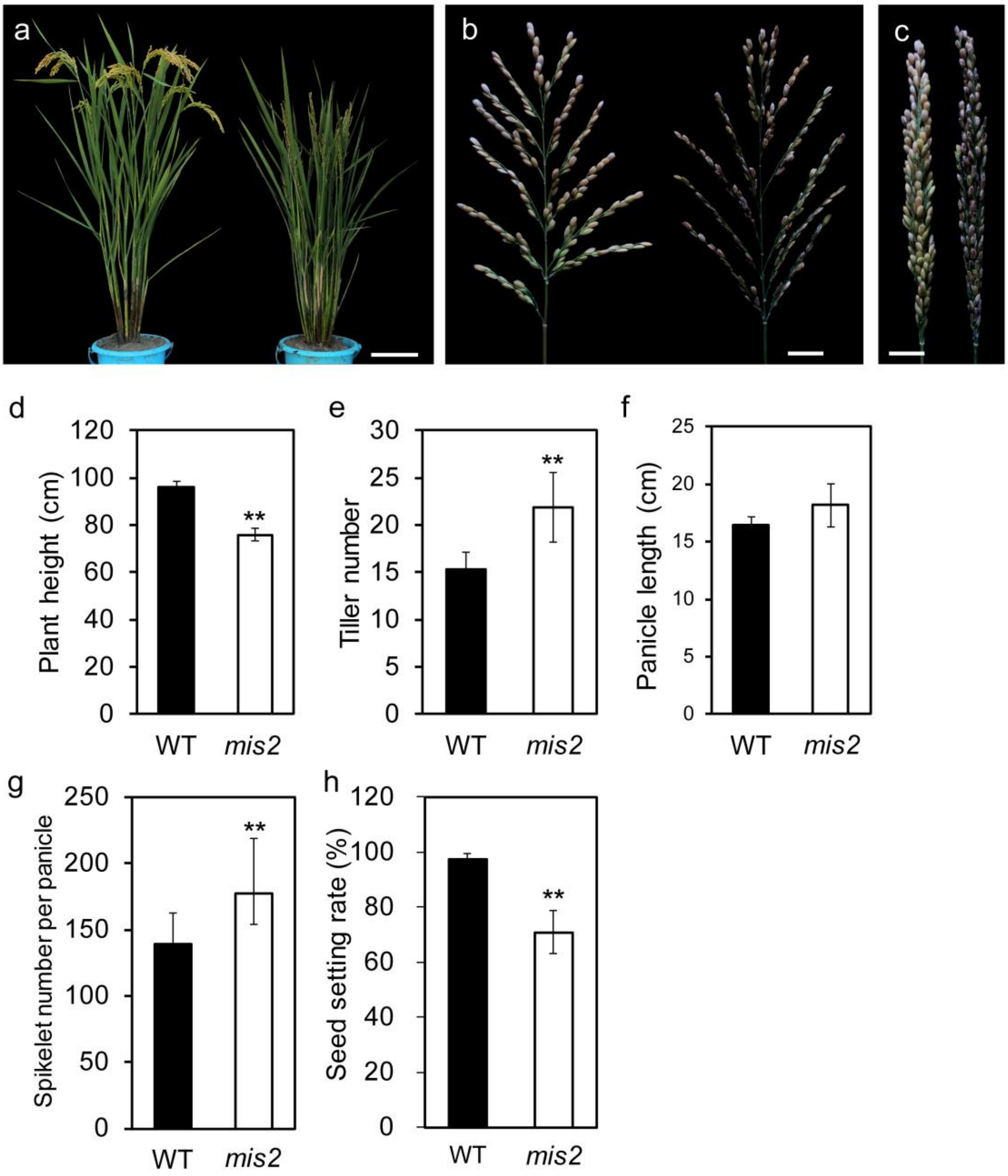

Figure S1. Phenotype of the *mi2* mutant plant.

(A) Whole plant phenotype at grain-filling stage. Bar = 20 cm.

(B, C) Panicle morphology at maturity stage. Bar = 2 cm.

(D-H) Quantification of plant height, tiller number, panicle length, spikelet number per panicle and seed setting rate.

** $P < 0.01$ by Student's t test. Data are given as mean ± SD ($n = 15$).

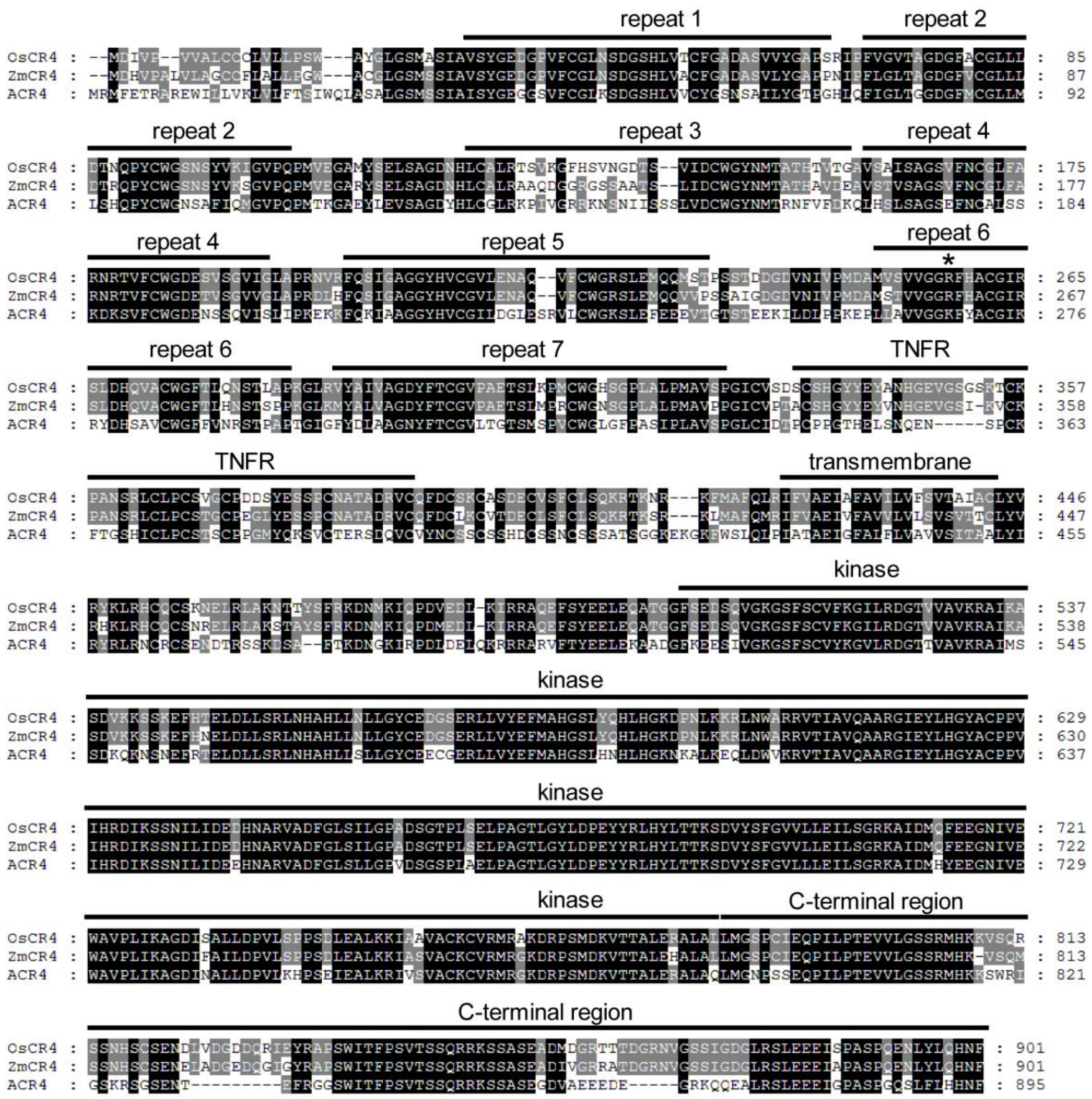

Figure S2. Sequence alignments of CR4 family protein from rice, maize and *Arabidopsis*.

The extracellular seven repeats, TNFR domain, transmembrane region, kinase domain and C-terminal region are underlined. The mutation in the *mis2* mutant is indicated by an asterisk.

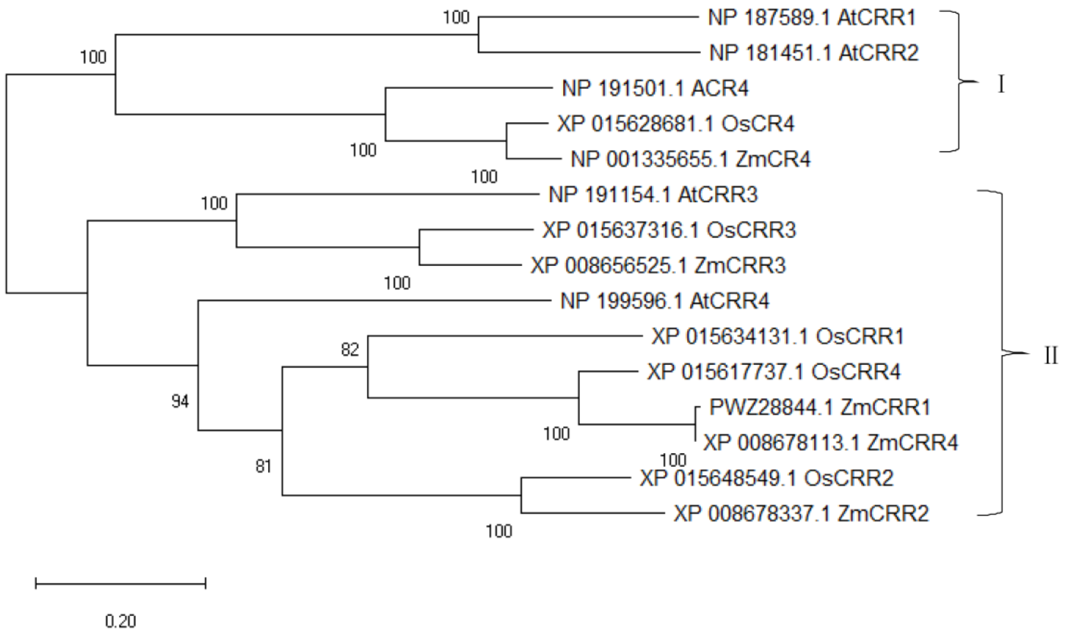

Figure S3. Phylogenetic analysis of CR4 family and CR4-related proteins.

Phylogenetic tree of CR4 family and CR4-related protein in rice, maize and *Arabidopsis*. The phylogenetic analysis was carried out by MEGA X version 10.0.1 with 1000 bootstrap replicates. The numbers on the branch sites indicate bootstrapping values. Accession numbers are shown. The tree was constructed using the distance method with maximum likelihood.

Table S1

Primers used in this study

Primer	Forward (5'→3')	Reverse (5'→3')
Molecular markers used for gene mapping		
M1	GCGAG ATGGG CAGCT ACTAC	ACACA ATGTC CAGCT TGCAG
M2	TTCGCTATCTCTTCCCGCTA	CTGTTTCGGACTGACTACAG
M3	TGCTGTCATCACTGGAATCT	ACGTGTTTGACCATTCGTCT
M4	AAGGTTAGGCGTGGATTCCTC	GAGATGAAGGAATGTTCAGTCC
M5	CACGCAGAGATAAGCTCAAC	GCTACAGTAACCCTCATGTGC
M6	CGAATTGAAGACGGGTGCAA	CCTCGAGGTCACCGTGTT
M7	AACGGACATAAGAGGATAGC	CGCAGTTCTTATGAACGGAC
M8	GGATGATATTGGCATCTTTGC	GCCCTATTATCAGGACTACC
M9	CCACTAGCATGACTATATGTGG	CCTGACTAAGGTGCTAATGA
M10	GCAGGAACGAGTTCGAGGAA	CGAATGCGCATGTAGGAGCA
M11	ACCAACTACGATTACCCACCT	AGGCCAGGGTACATGACAAA
M12	CACAGTTGGGTTGAAGTACG	ACTTACTTCCTCCGCATCTG
MIS2 gene full length DNA sequence amplification		
MIS2-SEQ1	ATTGGTATGCACCGAGAGAG	GGCACCCCACAAGTGAAGTA
MIS2-SEQ2	GAATGTGCGGTTCCAGTCTA	CTGATAGAGGGATCCATGAG
MIS2-SEQ3	GGCTTCTCAGAGGATTCACA	GCCAGGTACCATTGGATTTA
Primers for qPCR analysis		
qOsUbiq	CACCCTGGCTGACTACAACA	TTCTTCTTGCGGCAGTTGAC
qMIS2	AGAGGTCATCTAACCATTCGTG	CCTCTGAGATGAAGTCACACTT
qROC1	TCATGGATGTGAATCAATTCGC	AAACTCCATTGACATCACTTGC
qROC2	CATCATGACGCACGCCAA	CTCGACACGATGTTCGAGAA
qROC4	GGCCCTTCTCATGTCTTCTATT	AACTTCAACATGCTCCGTTTAC
qROC5	GTATCTTCACTCCTTCCTACCG	TGTTGTCGATGATGACTAGACC
qROC6	ATTTGTAGTGAGCTCGCGTGAT	CTACCGTCCATAGTTTGCAGG
qROC8	CAGATCAAGTTCTGGTTCCAGA	CTTCTGCTCGTCGAAGTAGTC

qGS5	CATTCCATGCAAATGCCAGTGG AC	CAGCCCTGCTTTGATGAGCTTG
qGW2	CAGCAGCGCATTCCCAGTTTTC	GTGGTCAGCCGAGCACTCTC
qSRS1	CCGAAATTAGGTACCGGTATCA	AATCAAACAACAGAGGAAACGC
qSRS3	AGCTTACAGAAGTTCTTCGTGA	CTTTTCTTGTATTGCTGCCCTT
qSRS5	TGACGAGTACTAGAGAGGTTCA	CAAAACCAGACACACAAACTGA
qGIF1	ATCACCAACGGGAAGATATCTC	GTTATTGAAAACGTAAAGGCGC
qBG1	GTACGAGACGACCAGAGTG	AGATATTCAGACAACTCTGGCG
qGSE5	GACATCGCCATGTATATGTTCG	GTACGACGACGACTCGATG
qFUWA	AGCAACATTGTGCGAATAACTC	CATCACTTGGTTTGCCATCTAT
qXIAO	GCTGCTGGTCTATGACTACAT	ATTGGCCAATTGAGAATGTGTC
qBRD1	TGGAGAAGAACATGGAATCACA	GGAATGTTGCAATTTCTACGGT
qBRD2	CTCTACAAGCTCCCTGTGAAAA	GTGAGAAGCATCAAACATCCTC
qD2	CCAACTGGAAGAGGAGAACATA	ACATGTAGTCTGTCCATTGCAA
qD11	CATCCATGCTTCTTGTTGTGAG	CTTACCTATAGCCTCACACCAG
qBU1	ACTTCACTTCAGTTCAGTAGGG	ATGAGATCAAGCTGGCTACTAC
qBZR1	ATTTGGGCGATTTCATTCTAGC	CGTGAATAAAATCAGCCGTGAT
qTUD1	TTGAGCTGCTCAATTCGATATG	GCAATTCTACTAGTTGCGGAAC
Primers for subcellular localization		
MIS2cDNA	CCATGATTACGAATTCATTCTTC CAATGCAGGGCTTC	CCCTTGCTCACCATGGTACCACAG AAGTTGTGCTGCAAGTACAG
Ara6CDS	GGACTCTTGACCATGGGATGTG CTTCTTCTCTTCC	TGCTCACCATGGATCCTGACGAAG GAGCAGGACGAGGTA
Primers for complementation construct		
MIS2gDNA	CCATGATTACGAATTCGTGACG GTGAGGTTATTTGTGCTC	GTCACCAATTCACACGTGGATGCA TGGTAAAGTAATCCAGGC
Primers for GUS construct		
MIS2prom	CCATGATTACGAATTCGTGACG GTGAGGTTATTTGTGCTC	CTCAGATCTACCATGGCAGGCACA ATGTCCATTTCTTG
